# Mapping the status of the North American beaver invasion in the Tierra del Fuego archipelago

**DOI:** 10.1371/journal.pone.0232057

**Published:** 2020-04-24

**Authors:** Alejandro Huertas Herrera, María Vanessa Lencinas, Mónica Toro Manríquez, Juan Andrés Miller, Guillermo Martínez Pastur

**Affiliations:** Centro Austral de Investigaciones Científicas (CADIC-CONICET), Ushuaia, Tierra del Fuego, Argentina; University of Potsdam, GERMANY

## Abstract

Quantifying the presence and environmental impact of invasive species is the starting point for research on management and nature conservation. North American beavers (*Castor canadensis*) were introduced to Argentina from Canada in 1946, and the species has been identified as a major agent of environmental change in the Tierra del Fuego archipelago in the Anthropocene. We studied the invasion status (distribution and density) of beavers through analyses of the dam densities in the Tierra del Fuego landscapes. We identified beaver dams with a GIS using visual interpretation of high-resolution aerial imagery from Microsoft Bing, Google Earth and HERE and related them to natural environmental gradients. These factors comprised geographic (vegetation zones and distance to streams), climatic (temperature, precipitation, evapotranspiration and net primary productivity) and topographic (elevation and slope) data. The datasets (dams and factors) were combined, and the data from the different zonation classes were subsequently compared using ANOVAs and Tukey’s mean comparison tests. Deviations from the mean density (*x* mean density—*x* total mean density) were calculated to visualize the deviations for the studied factors. The datasets were also evaluated using principal component analyses (PCA). Our results showed a total of 206,203 beaver dams (100,951 in Argentina and 105,252 in Chile) in the study area (73,000 km^2^). The main island of Tierra del Fuego presented a greater degree of invasion (73.6% of the total study area) than the rest of the archipelago, especially in areas covered by mixed-evergreen and deciduous forests. The studied geographic, climatic and topographic factors showed positive trends (higher beaver preference) with beaver spread, which were all significant (p <0.05) when compared across the landscape. Although beavers are flexible in their habitat use, our empirical records showed that they had marked preferences and were positively influenced by the most productive forests. Here, we describe a scientific panorama that identified the drivers of species invasion based on satellite data and the available ecological datasets. The identification of such drivers could be useful for developing new tools for management and/or control strategies of the beavers in the Tierra del Fuego archipelago.

## Introduction

North American beavers (*Castor canadensis*) have been recognized as ecosystem engineers [1]. They build dams in riparian forests or where suitable aquatic environments prevail [2], mainly using the branches and trunks of trees [3], which change the stream hydrology, plant assembly and soil biogeochemistry, both at the local and landscape levels [4, 5]. When beavers reach maturity, they are forced to leave the family group [6]. Therefore, beavers often move to another area to establish a new pond and colony (e.g., in areas with low beaver densities) [7]. Beavers were introduced from Canada to Argentina on the main island of Tierra del Fuego in 1946 to develop a potential fur industry, which was later became unsuccessful [8]. Since then, they have spread throughout the Tierra del Fuego archipelago [9], nearly reaching the Cape Horn islands [10] and reaching the mainland near the city of Punta Arenas [7]. The availability of water and wood resources and the capacity of the beavers to change habitat structure, the high reproductive rate, and the lack of natural predators have contributed to the growth of the beaver populations [11, 12].

Currently, beavers are established in the foremost vegetation zones, occupying ecosystems from deciduous and evergreen *Nothofagus* forests to open ecosystems (e.g., grasslands and shrublands in the steppe), with elevations that vary from sea level to above the tree line (~800 m.a.s.l.) [12], causing the most significant alterations to the sub-Antarctic ecosystems in the Anthropocene [7]. For example, the flooded areas near a dam are converted into meadows that appear to be a long-term stable state (lacking signs of resilience), where many native species are not capable of surviving due to beaver-created gradients in soil moisture, light availability and herbaceous plant communities [5, 13]. Because of these significant impacts, beaver invasion is considered a major threat to conservation priorities [8, 14].

Species distribution data are essential for invasion science applications (e.g., potential distributions of species) [15, 16]. This information can be a tool for mapping and prioritizing sites for conservation [17]. In fact, the estimation of the presence of an invasive species is a starting point to assist with the location of eradication or control efforts [14, 18]. Although previous attempts have been directed towards the analyses of the impact of beaver invasion, its distribution, density and environmental thresholds have not been addressed spatially explicitly across the Tierra del Fuego archipelago. The aim of this study was to analyze the beaver invasion status and to relate this pattern with different factors at the landscape level. These factors comprised geographic (vegetation zones and distance to streams), climatic (temperature, precipitation, evapotranspiration and net primary productivity) and topographic (elevation and slope) environmental gradients. Complementarily, we define the following questions for this study: (1) Which is the magnitude (number and density of dams as a proxy) of the beaver invasion across the archipelago? (2) What are the optimal environmental conditions for beaver invasion? (3) How does beaver dam density vary across environmental gradients? This study might contribute to identifying the drivers of species invasion based on available satellite data and geospatial datasets (e.g., Google Earth Engine), allowing the development of new tools for the management and/or control of beavers in the Tierra del Fuego archipelago.

## Methods

### Study area

**Fig 1:**
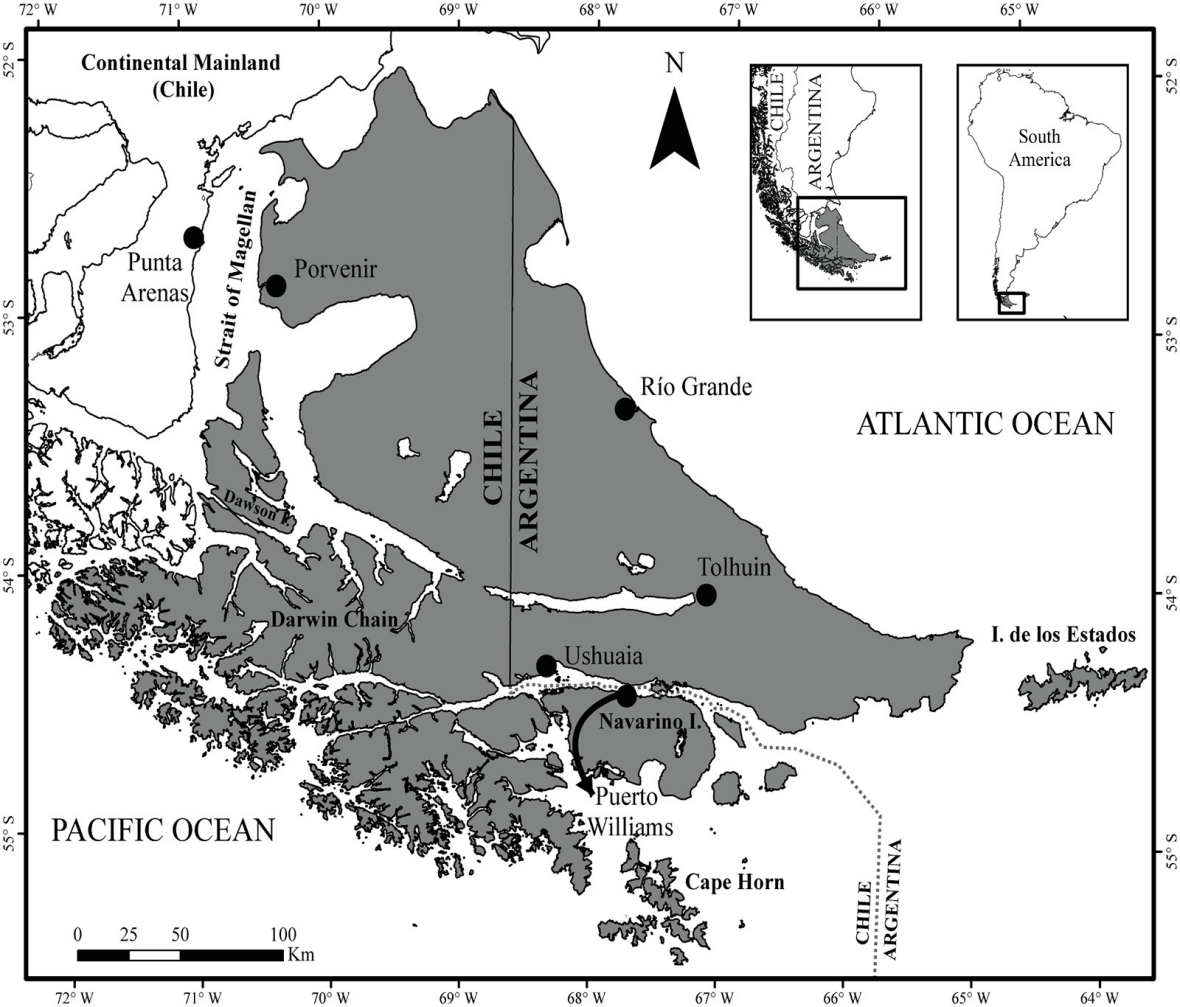
Study area. Archipelago of Tierra del Fuego showing the study area (dark gray) and the main cities in Argentina and Chile.

The Tierra del Fuego archipelago is located in the southern part of South America (52°-56° SL to 72°-63° WL, Fig 1) and is shared between Argentina and Chile. It covers approximately 73,000 km^2^, of which the main island of Tierra del Fuego occupies an area of 48,100 km^2^. The vegetation zones range from open ecosystems such as grasslands, shrublands and peatlands to woody habitats such as temperate deciduous and evergreen forests [19]. Forests cover approximately 50% of the land and are mainly composed of *Nothofagus* species: two deciduous species (*N*. *antarctica* and *N*. *pumilio*) and one evergreen species (*N*. *betuloides*) [20]. The archipelago includes numerous islands and fjords, where the Andes Mountains define the regional relief and climate [21]. The elevation ranges from 0 to 2,488 m.a.s.l. Here, glaciers and peatlands play an essential role in hydrological regulation, and small streams carry surface water into the main rivers [22]. The climate is characterized by short cool summers and long snowy winters with frequent occurrence of frosts [19]. The precipitation (including drizzles, rain, sleet, snow, graupels and hail) gradient increases from north to south; it is less than 300 mm year^-1^ in the steppe, while in the south, it exceeds 2000 mm year^-1^ [23]. The mean temperatures vary in winter (July and August are the coldest months) from -4°C to 3°C, and during summer (January and February are the warmest months) average from 8°C to 11°C [19]. With a diverse geography and relatively low-density human population, this archipelago is considered one of the last regions with intact ecosystems in the world [24] and comprises numerous national parks and provincial reserves belonging to the ecological heritage of humankind (e.g., the Cape Horn Biosphere Reserve). The main inhabited areas are located on the seashore: the cities of Río Grande and Ushuaia in Argentina and the towns of Porvenir and Puerto Williams towns in Chile.

### Remotely sensed beaver data

**Fig 2:**
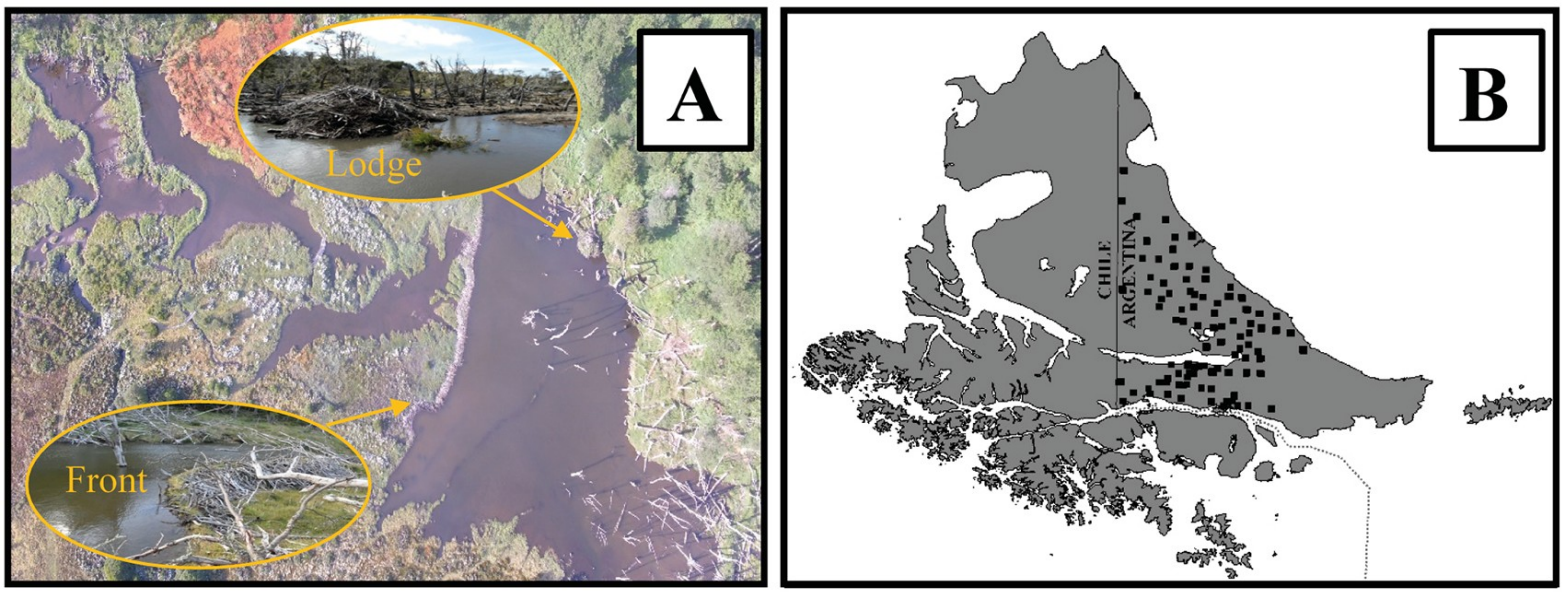
Remotely sensed beaver dam data. (A) Example of the analyzed beaver dams (photo by A. Huertas Herrera). (B) Verification plot (black quadrants) distribution in the Argentine portion of Tierra del Fuego.

We used high-resolution aerial imagery from Bing aerial maps (Microsoft, USA), Google Earth (Google, USA) and HERE (HERE Technologies, Netherlands) for the entire archipelago to determine the occurrence of beaver dams (abandoned dams with meadows and active dams with ponds) as proxies of beaver invasion data (i.e., to detect the animal tracks and potential habitat expressed by dams). We verified the availability and quality of images because many areas presented frequent cloud cover, and among them, we selected the most recent images. The temporal resolution of the image set varied from January 2002 (which mainly had imagery of remote areas) to December 2019 (which mainly had imagery from the main island). Active and inactive beaver dams were distinguished through the recognition of the flood area (front and tail), evident tree removal, and presence of lodge structures, beaver marshes or stream channel structures (Fig 2A). Additionally, it was possible to observe the multitemporal changes with the image history provided by Google Earth, and this was used to improve the detection capacity of the dams. We visually examined the “true color” of the large (e.g., ± 2 ha) and small (e.g., ± 50 m^2^) dams throughout the entire archipelago by using the basemap available in ArcGIS 10.3 (ESRI, USA). Then, ~75,000 sampling plots of 100 ha each were created from north (52°) to south (56°) and on a latitudinal gradient in the west (72°) to the east (63°) direction. The detected dams were manually marked by clicking on the basemap at a scale of 1:4,000, and the created points were stored in a point shapefile. This shapefile (a point cloud) was used to estimate the number of dams and generate a 30-meter resolution raster of the dam density at 1 km^2^ by using the Kernel Density tool in ArcGIS 10.3.

Field training plots (verification plots) were created in the Argentinean part of Tierra del Fuego because of their accessibility (Fig 2B); some were assess with the use of a Phantom 4 drone (Sensor 1/2.3 CMOS, 12.4 MP photos) (Fig 2A). We checked the beaver dam detection in 72 verification plots (quadrants of 1 km^2^) to figure the error that resulted from the visually counted beaver dam data (abandoned and active dams). We used a drone to reach some inaccessible areas (e.g., rocky areas and deep valleys), especially in the autumn season when the weather was favorable (e.g., no windy or freezing days). We constructed a confusion matrix to calculate the error in beaver dam counting based upon the differences between the marked visual survey and the field-verified dam locations (including false negatives and false positives). The remote survey underestimated the detected beaver dams by 11.7% (S1 Table), where the main errors occurred in the visual identification of abandoned beaver dams from the imagery (75.0%).

### Geoprocessing and analyses of the environmental dataset

**Fig 3:**
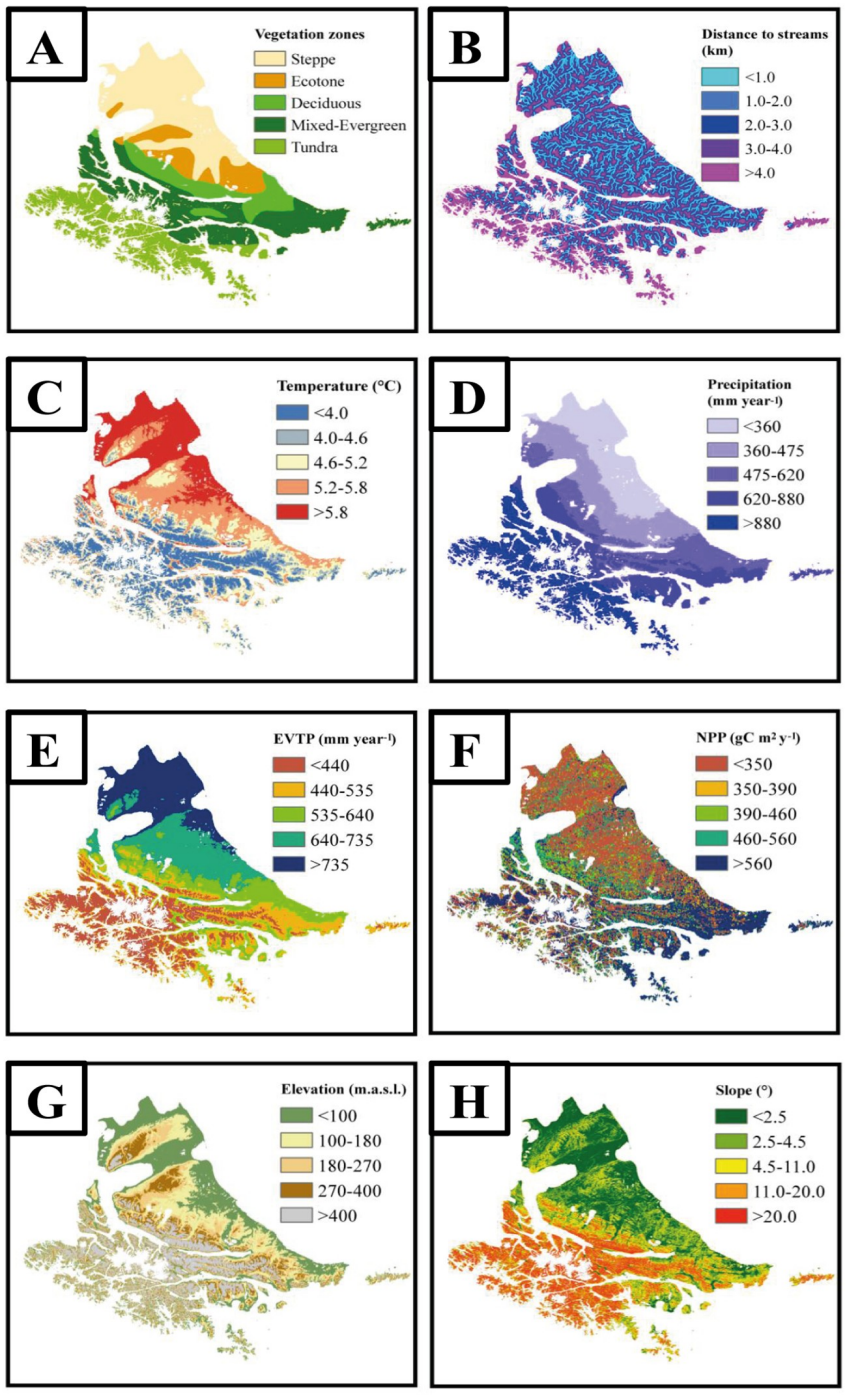
Distribution of the analyzed geographic (A-B), climatic (C-F) and topographic (G-H) factors on the Tierra del Fuego archipelago. (A) Vegetation zones were obtained from Borromei et al. (2014). (B) Distance to streams was obtained from https://earthengine.google.com/. (C) Temperature and (D) precipitation were obtained from http://www.worldclim.org/version2. (E) Evapotranspiration (EVTP) was obtained from http://www.cgiarcsi.community/. (F) Net primary productivity (NPP) was obtained from http://files.ntsg.umt.edu/. (G) Elevation and (H) slope were obtained from https://earthengine.google.com/.

The environmental dataset consisted of geographic, climatic and topographic factors (Fig 3), all of which were in raster format at 30-meter resolution and projected into the World Geodetic System 1984 (WGS 84) coordinate system. The factors were chosen because they were presumed to be associated with the impact [12, 13], potential distribution [7, 9, 12, 14] and habitat use of beavers [7, 11, 14]. The analyzed geographic factors included the archipelago’s main vegetation zones and the proximity to streams. The vegetation zones were digitalized from Borromei et al. [25] and were categorized as steppe, forest-steppe ecotone (henceforth ecotone), deciduous forests, mixed-evergreen forests and tundra. The proximity of the active and abandoned dam to streams was calculated using the stream shapefile obtained from the HydroSHEDS river network at 15-second resolution (https://earthengine.google.com/). The stream proximity value was calculated by Euclidian distance to the stream. The climatic factors included the modeling of temperature (°C) and precipitation (mm year^-1^) for the 1970–2000 period. These datasets were taken from WorldClim (http://www.worldclim.org/version2). We also used global aridity and global potential evapotranspiration for the same period [26], which were obtained from the Consortium for Spatial Information (CGIAR-CSI) (http://www.cgiarcsi.community/), and the net primary productivity (NPP) of the vegetated land surface. The NPP was derived from MODIS products (MOD17A3), and the annual NPP (gC m^2^ yr^-1^) for 2016 was downloaded from the Numerical Terradynamic Simulation Group (NTSG) public data repository (http://files.ntsg.umt.edu/). This raster was multiplied by 0.1 to obtain the actual value, and the NPP data for covers such as lakes or cities (fill value 65,535) were excluded because these do not correspond to the vegetated land surface. The studied topographic factors included the elevation and slope, and both were derived from the digital elevation model (DEM) generated by the Shuttle Radar Topography Mission (SRTM). This dataset was obtained from the Google Earth Engine (https://earthengine.google.com/) (Google, USA). The slope was calculated using the DEM and the Surface toolset in the Spatial Analyst toolbox in ArcGIS 10.3. The slope represented the rate of change in elevation calculated in degree units, where the range of slope values varied between 0 and 90 degrees.

To match the dam density with the environmental factors, we employed the Combine tool in ArcGIS 10.3. To standardize the raster, the archipelago boundary was taken from OpenStreetMap and was employed as a mask for all environmental datasets (https://planet.openstreetmap.org/); glaciers and lakes (>10 ha) were also classified as no data. The combined attribute table of the resulting grids was used to characterize and determine the even levels of each analyzed geographic, climatic and topographic factor based on the number of pixels (30 meters) relative to the total of the extracted values. This was done so that the levels for each factor included the same number of beaver dam density values per proportion of the surface so that the density values were not lower because there were fewer surfaces. Five levels of each variable were considered per factor to determine the species ecological preferences. In the continuous variables (distance to streams, temperature, precipitation, EVTP, NPP, elevation, and slope), each category was represented by 20% of the total area of the archipelago, while in the discrete variables (vegetation zones), we used the effective area occupied by each category.

A grid of 567 hexagons (each hexagon = 10,000 ha) that covered the entire archipelago was created in the GIS. The hexagons were used as sample units to estimate the beaver dam density and to evaluate its variation according to environmental factors. For this, five levels for each geographic, climatic and topographic factor were defined to follow the procedure of the previous characterization, where the number of pixels for each factor and the beaver dam density values were extracted. The beaver dam density values (mean ± standard error) were extracted for each level of the analyzed factors and assigned to the hexagons. For the hexagonal binning processing, we established a minimum threshold of 25 hexagons for each level of the studied factor to define a suitable sampling size for the statistical analyses (Table 1). All raster statistics were calculated using the Zonal Statistics tool in ArcGIS 10.3.

**Table 1:** Hexagon quantity (total of 567 for the whole study area) obtained for each level of the analyzed geographic, climatic and topographic factors.

Factors	Type	Level	*n*
Geographic	Vegetation zones	Steppe	125
		Ecotone	58
		Deciduous	201
		Mixed-evergreen	27
		Tundra	156
	Distance to streams	<1.0	210
		1.0–2.0	168
		2.0–3.0	102
		3.0–4.0	30
		>4.0	57
Climatic	Temperature	<4.0	116
		4.0–4.6	113
		4.6–5.2	113
		5.2–5.8	113
		>5.8	112
	Precipitation	<360	106
		360–475	99
		475–620	105
		620–880	126
		>880	131
	Evapotranspiration	<440	110
		440–535	148
		535–640	95
		640–735	103
		>735	111
	Net primary productivity	<350	33
		350–390	79
		390–460	135
		460–560	146
		>560	174
Topographic	Elevation	<100	121
		100–180	147
		180–270	123
		270–400	84
		>400	92
	Slope	<2.5	84
		2.5–4.5	63
		4.5–11.0	115
		11.0–20.0	121
		>20.0	184

### Data analyses

The beaver dam densities associated with each level of each studied factor were expressed as the mean ± standard error. The statistical significance was assessed by one-way ANOVA analyses. Data normality and homoscedasticity were verified with the Shapiro-Wilk and Levene tests, respectively. The beaver dam density was log-transformed to meet the assumption of normality, but the non-transformed averages are presented. We used a post hoc Tukey’s test (p <0.05) for further mean comparisons. Next, we calculated the deviations from the mean beaver dam density (*x* mean density—*x* mean total density) to visualize the variations in each environmental factor at the landscape level, e.g., negative responses indicated a decrease in the beaver dam density (e.g., lower beaver preference), while a positive response indicated an increase (e.g., higher beaver preference). All analyses were conducted using Statgraphics software (Statistical Graphics Corp., USA).

We also performed a principal component analysis (PCA) to explore multivariate relationships between dam density and the measured environmental variables (distance to streams, temperature, precipitation, EVTP, NPP, elevation and slope). We classified the plots according to (i) the density of beaver dams considering four categories: not invaded (zero dams), low density (1 to 5 dams), middle density (5 to 15 dams) and high density (> 15 dams); and (ii) the vegetation zones (steppe, ecotone, deciduous, mixed-evergreen, tundra). The PCA analysis included a Monte Carlo permutation test (n = 999) to assess the significance of each axis. We selected variance/covariance-centered coefficients among the columns to obtain the cross-products matrix. The calculated scores for the columns were distance-based matrix biplots. We conducted a PCA in PCORD version 5.01.

## Results

**Fig 4:**
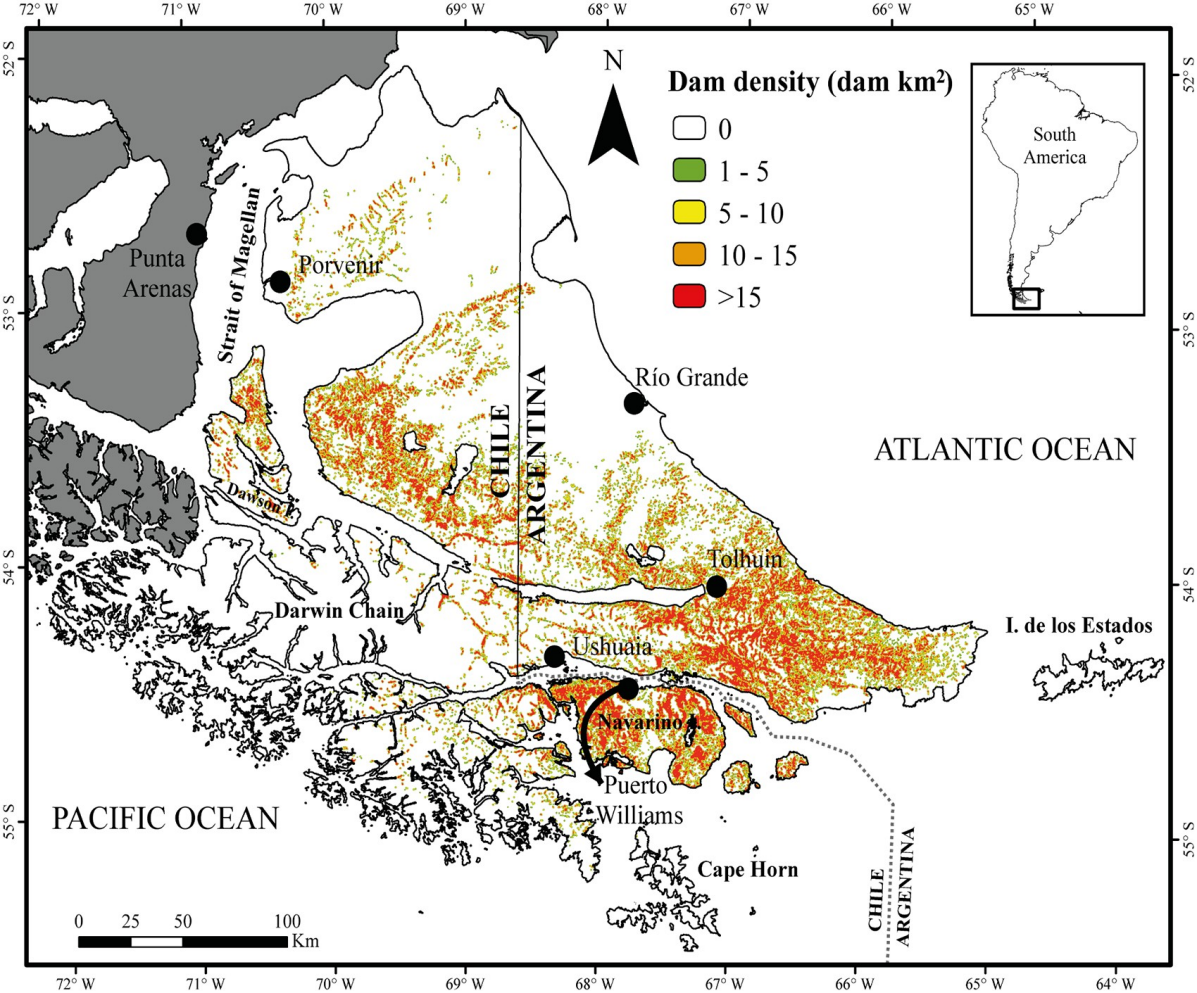
Beaver dam density (dams per km^2^) in a search radius of 1 km^2^. The zone that was not analyzed is in gray.

The beaver dam density map showed that the administrative border between Argentina and Chile does not influence the species distribution (Fig 4). Our study revealed a total of 206,203 dams, with 100,951 in Argentina and 105,252 in Chile (image analysis data for the entire image mosaic ranged from 2012 to 2019). As expected, the main and largest island presented most of the beaver dams (73.6% of the total dams). Among the smaller islands (26.4% of the total study area), the islands of Navarino (36,624 dams) and Dawson (7,115 dams) in Chile were the most affected by the beavers. In contrast, Isla de los Estados in Argentina and multiple islands of the southwestern Darwin Chain in Chile appeared to have not yet been invaded. The mixed-evergreen forests (103,947 dams) and deciduous forests (55,843 dams) were the most extensively invaded among the defined vegetation zones (50.4% and 27.1% of the total beaver dams, respectively), whereas the ecotone (19,379 dams) was the least affected (9.4%). In addition, extreme environments without forests also presented signs of beaver presence, e.g., 6.2% (12,707 dams) of the beaver dams were found in the steppe and 6.9% in the tundra (14,327 dams) environments.

**Fig 5:**
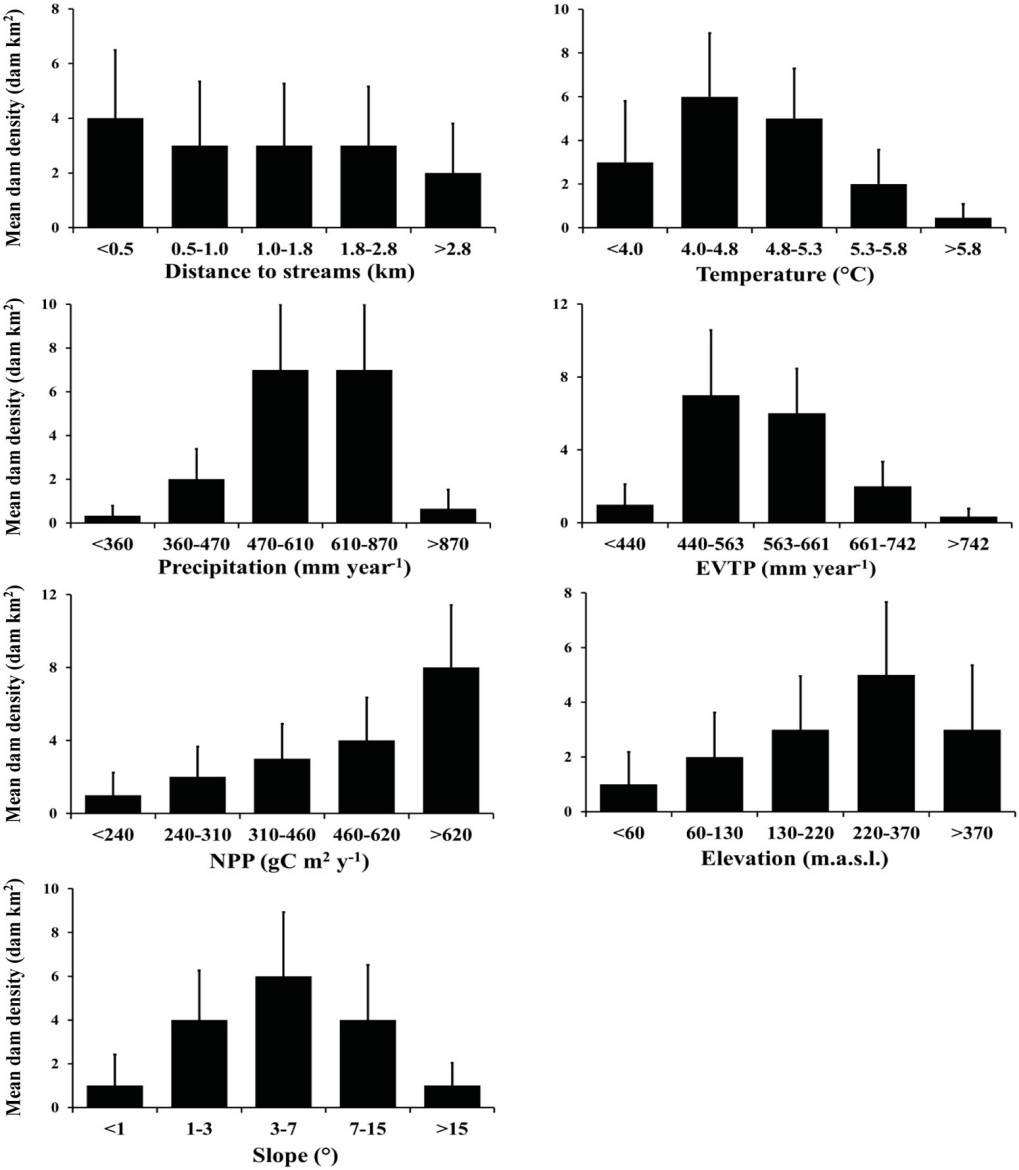
Mean beaver dam densities (dam per km^2^) at different levels of each analyzed geographic, climatic and topographic factor. Error bars represent the standard deviation. NPP = net primary productivity, EVTP = evapotranspiration.

The analyses that compared the different levels of climatic and topographic factors showed that the beavers inhabited a wide range of environmental conditions (Fig 5), but their preferences differed. A decrease in the mean beaver dam density was shown as the distance to the streams increased (from <0.5 km to 2.8 km, 3.9 to 3.7 dams km^2^), with a decrease in the density at a distance greater than 2.8 km (2.5 dams km^-2^). The annual mean temperature between 4.0°C and 5.3°C was the optimal range (reaching up to 5.9 dams per km^-2^), which represented 60% of the beaver dams. Likewise, beaver dams were less abundant in areas with low (<470 mm yr^-1^) or high (>870 mm year^-1^) precipitation (less than 2.2 dams km^-2^), where the optimal areas received between 470 and 870 mm year^-1^ (~7.3 dams km^-2^). In coincidence with these observations, the optimal range of potential evapotranspiration for beaver dam occurrence was between 440 and 661 mm year^-1^ (~7.4 dams km^-2^). The analysis of NPP showed that the highest densities of beaver dams were in the most productive areas of the archipelago (>620 gC m^2^ yr^-1^, 7.8 dams km^-2^). Regarding topography, beaver dam density increased with elevation, with a maximum at 220–370 m.a.s.l. (reaching 5.5 dams km^-2^) and in areas with lower to middle slope values (3° to 7°, 5.9 dams km^-2^).

**Fig 6:**
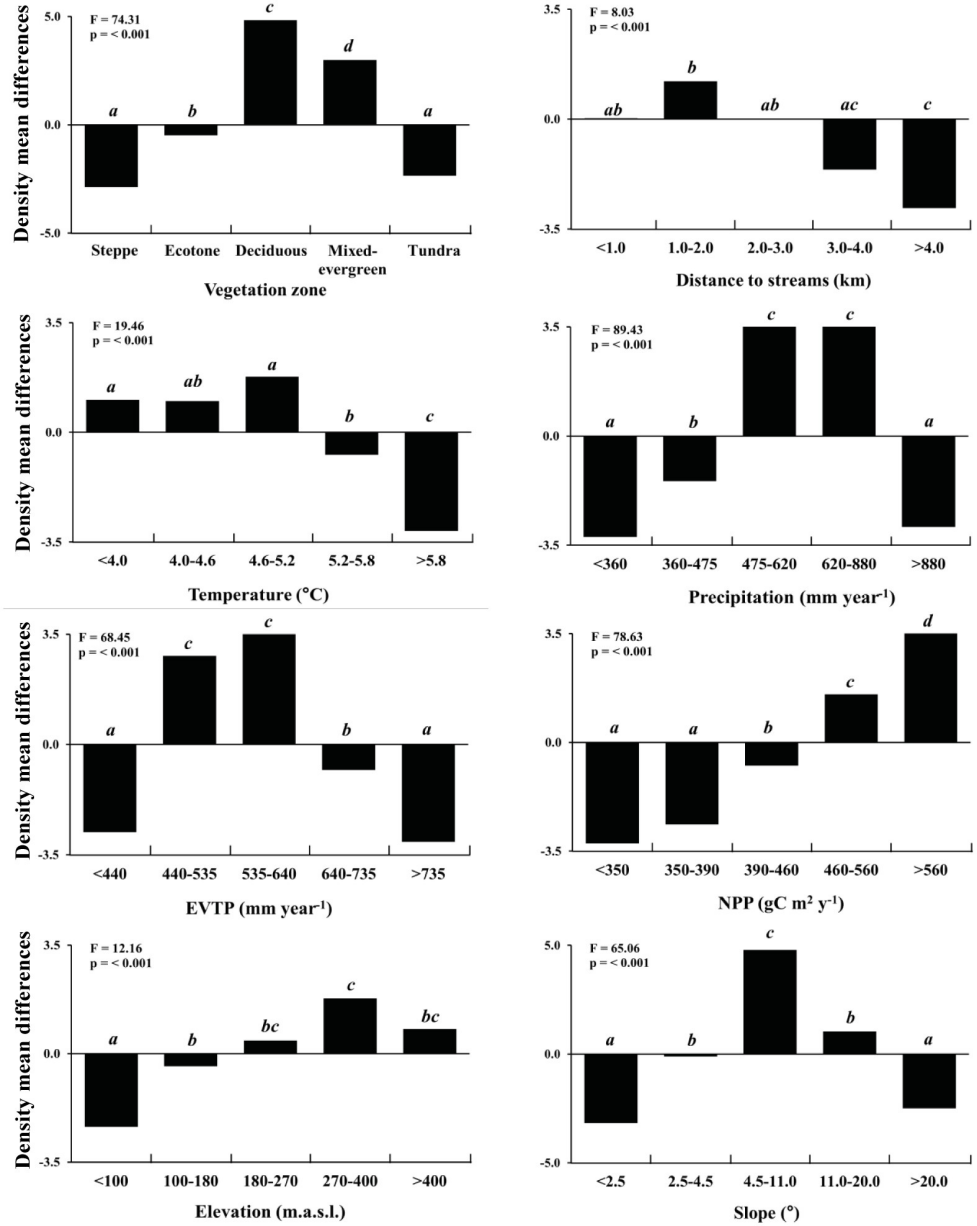
ANOVAs for the mean density differences (dams per km^2^) comparing the analyzed geographic, climatic and topographic factors. Different letters indicate significant differences between the levels (Tukey’s test at p <0.05). NPP = net primary productivity, EVTP = evapotranspiration.

The ANOVAs of the comparison of mean deviations showed significant differences for all studied factors (Fig 6). For the vegetation zones, positive relationships were found with the deciduous (+4.8 dams km^-2^) and mixed-evergreen (+2.9 dams km^-2^) forests, and negative relationships were found with the ecotone (-0.5 dams km^-2^), steppe (-2.9 dams km^-2^) and tundra (-2.3 dams km^-2^). Notably, ecotones can be considered a distinctive group that tend to resemble open ecosystems (steppe, tundra) more than forests. For distances to streams of <1.0 km, the beaver density was almost equal to the total mean after that response ratios increased from 1.0–2.0 km (1.2 dams km^-2^) and then markedly decreased at distances higher than 3.0 km (from -1.6 to -2.8 dams km^-2^; 40% of the area). Temperature presented two distinct groups; one group was positive at lower values (<5.2°C, +1.0 to +1.8 dams km^-2^), which represented 60% of the studied area, and the other group was negative at higher values (>5.2°C, -0.7 to -3.1 dams km^-2^), which represented the other 40%. Precipitation showed negative trends in 60% of the studied area (-1.6 to -3.2 dams km^-2^ in arid areas with <475 mm yr^-1^ and ~3.5 dams km^-2^ in humid areas with >880 mm yr^-1^) and a positive trend between 474 and 880 mm year^-1^ (40% of the area). Similarly, negative trends were found in 60% of the area for the EVTP (<440 and >640 mm yr^-1^, -0.8 to -3.0 dams km^-2^), while positive trends occurred at values between 440 and 640 mm yr^-1^ (+2.8 to +3.5 dams km^-2^). The trend with NPP was negative in 60% of the area (<460 gC m^2^ yr^-1^, -0.7 to -3.2 dams km^-2^) and positive for the other 40% (>460 gC m^2^ yr^-1^, +1.5 to +3.5 dams km^-2^). The elevation indicated that areas below 180 m.a.s.l. presented adverse conditions (40% of the area) (-0.4 to -2.3 dams km^-2^), while in the rest of the area, the trend was positive (>270 m.a.s.l.) (+0.4 to +1.8 dams km^-2^). For the slopes with lower (<4.5°) and higher values (>20.0°), the trends were negative (-0.1 to -3.2 dams km^-2^), representing 60% of the area, and the other areas presented positive values (+1.0 to +3.5 dams km^-2^) between 4.5° and 20.0°.

**Fig 7:**
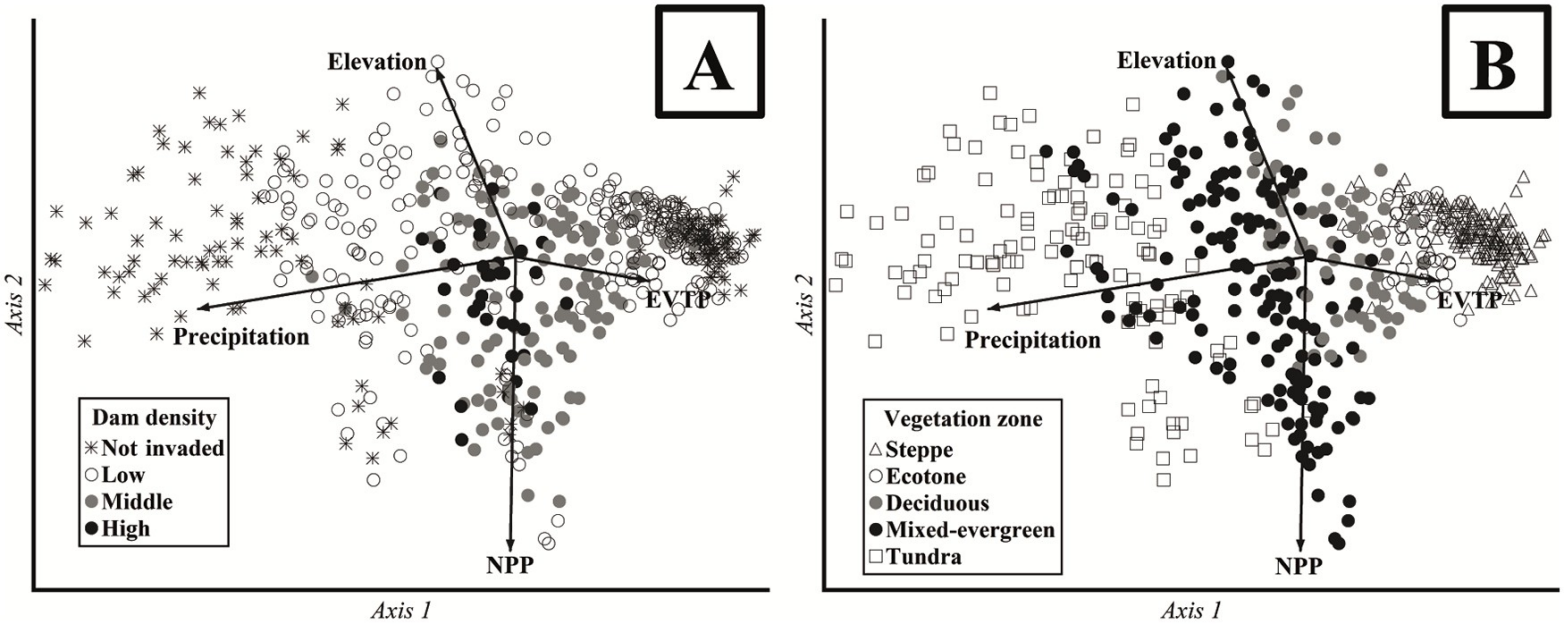
Principal components analysis (PCA) using the analyzed geographic, climatic and topographic factors. The plots were classified according to (A) beaver dam density (dams per km^2^) considering the not invaded (zero dams), low density (1 to 5 dams), middle density (5 to 15 dams) and high density (> 15 dams) areas; and (B) the vegetation zones (steppe, ecotone, deciduous, mixed-evergreen, tundra). Distance to streams, temperature and slope were not graphically represented. NPP = net primary productivity, EVTP = evapotranspiration.

The PCA analyses highlighted the influence of some variables (precipitation, EVTP, NPP and elevation) over others (distance to streams, temperature and slope) (Figs 5 and 6). The first three axes explained 70.2% (p = 0.001), 15.1% (p = 0.535) and 13.7% (p = 0.851) of the variation in the total dataset. Axis 1 was mostly related to precipitation (eigenvector -0.898) and EVTP (eigenvector 0.376), while Axis 2 was more related to NPP (eigenvector -0.829) and elevation (0.537 eigenvector). The distance to streams (Axis 1 = -0.003, Axis 2 = -0.006), temperature (Axis 1 = 0.002, Axis 2 = -0.001) and slope (Axis 1 = -0.020, Axis 2 = 0.0134) were the variables that contributed the least to plot variation. Therefore, we excluded these factors (distance to streams, temperature and slope, which had absolute values lower than 0.020 in Axes 1 and Axes 2) in the PCA graphic to simplify and clarify the interpretation. When the density classification was used (Fig 7A), the plots with middle and high dam density were grouped in the center, where the variables showed their lower absolute values (e.g., low elevation values). Meanwhile, the higher absolute values of each variable were related to lower dam density categories (e.g., precipitation or elevation). Moreover, extreme values of precipitation and EVTP were correlated with the not invaded and low dam density categories. When plots were classified for the vegetation zones (Fig 7B), the middle and high dam densities were mostly found in mixed and deciduous forests and to a lesser extent in the ecotone, whereas the steppe and tundra showed most of the not invaded areas or low dam densities.

## Discussion

### Magnitude of beaver invasion in the archipelago

The status of the beaver invasion was proportionally similar between Argentina and Chile (~50% of the dams in each country), where nearly 50% of the archipelago had been invaded until today. Approximately 200,000 dams were distributed across the different environmental gradients (Fig 3), and the low (e.g., <5 dams km^-2^) and high (e.g., >15 dams km^-2^) density values provided an essential clue that there was an underlying natural process that was needed to understand the invasion process. The main island hosted higher dam densities, probably because it was the area where the first individuals were released [7] and because this island has a larger surface and more forested environments than in the southwestern Darwin Chain and the Isla de los Estados, allowing it to support greater beaver populations. Nonetheless, previous work recognized the potential for invasion to the continental lands or other areas of the archipelago that have not been invaded to date [11, 14, 27]. Undoubtedly, the mixed-evergreen and deciduous forests were the most occupied vegetation zones, while the less affected areas were found in the ecotone, dry steppe and tundra.

A possible explanation for the higher beaver dam density in those forested ecosystems might be that beavers require tree inputs to build their lodges and dams and preferentially use forest riparian woody material [28]. However, it is interesting that the open ecosystems (e.g., steppe) also had the presence of beaver, which reflects the plasticity of the species in non-woody ecosystems. In these areas, the beaver used woody materials from shrubs, half shrubs, woody cushion plants and other perennial plants [29]. Earlier studies in the archipelago have indicated that this invasion caused the most extensive alteration to these vegetation zones [12, 29, 30, 31], where deciduous *N*. *pumilio* was more desirable to the beavers than *N*. *antarctica* and evergreen *N*. *betuloides* [32]. Because beavers can quickly build dams or recolonize abandoned dams in a short time [4,29], the rapid construction of dams is critical to consider because our verification plots detected an underestimation of beaver presence (~12%). Similar to Henn et al. [12], this underestimation was mostly because abandoned beaver dams become permanent grasslands that cover the dam itself due to the small size of the dams, or dams may be built below tree cover, where they are not detected. In addition, new dams could be built between the satellite image date and the field verification date. A periodic (e.g., monthly or annual) comparison of images (in summer and winter season) could be useful for detecting dams across distinct vegetation zones (e.g., using distinct bands of the spectrum). Unfortunately, the Bing, Google Earth and HERE image sets are from different periods, which is currently an inherent problem of this mapping strategy [16]. Thus, some pristine areas might be already invaded, or some other might have a higher density of dams (e.g., from 2012–2019). Indeed, imagery such as Landsat 8 (30-meter resolution) or Sentinel-2A (10-meter resolution) exists that has an excellent temporal resolution (e.g., monthly or weekly) for testing the potential for beaver dam discrimination (e.g., available in Google Earth Engine). However, these images are not practical for detailed identification [33], and because the pixel size may include several small dams, the digital value may be averaged in other covers, such as groundwater or grassland. In this context, the satellite images provided by Bing or Google Earth have been useful for studying the landscape-level presence of beaver dams in remote or difficult-to-access places [13, 31, 34]. These approaches have confirmed these images as powerful tools for ecological science applications [16]. For instance, earlier works (e.g., 11, 12) have focused on analyzing the impact and habitat use of beavers (e.g., affected cutting surface) at the landscape level using a single satellite data source. The beaver impacts and habitat use were associated with environmental factors, but the areas that have been invaded (e.g., the main island of Tierra del Fuego) and those that have not (e.g., several other islands of the Archipelago) were compared. We focused on testing the beaver invasion status (e.g., the spread of beavers to show the drivers of species invasion), through species environmental preferences of both invaded and noninvaded areas. Regarding the method, earlier works used mainly Google Earth to delimit the modifications to the landscape, whereas we used different satellite sources to study the presence and density of dams in the entire territory, which may help to create habitat suitability maps and allow extrapolation and refinement models in other important areas (e.g., noninvaded areas).

### Optimal environmental conditions for beaver invasion

Beavers occupy a wide range of environmental conditions. Although they are flexible in their habitat use, our empirical records (with density as a surrogate) showed that they had marked preferences. Previous studies have indicated that the original invasion was more rapid since there were several optimal conditions guaranteed by the pristine nature [7, 11]. Of course, the environmental conditions for their introduction were deliberately selected by humans for their rapid population growth to sustain the fur industry [8]. In the Tierra del Fuego, the vegetation zones are made up of many waterways and small streams that make up complex network of water basins, where the beaver presence has been correlated to the distance to the stream [7, 28, 35]. A greater preference was found for distances from 1–2 km and not less than 1 km. A possible explanation for these results may be the details of the processed dendritic drainage pattern. In this study, the main watercourses and streams were considered, but these could have small peripheral streams with the presence of beavers that were not available in the shapefile used for the analysis.

We also found other conditions where beavers were likely to live, being positively influenced by the highest productive forested vegetation zones (e.g., NPP). As mentioned, they preferred to forage and build dams in riparian forests (mainly *N*. *pumilio*), but other highly productive non-forest ecosystems exist within the vegetation zones where beavers can live (e.g., peatlands), which combines their environmental preferences (e.g., water) and food requirements (e.g., riparian forests). Thus, these environments rarely occurred at elevations between 130–370 m.a.s.l. and over ~800 m.a.s.l. The invasion can be understood primarily due to the presence of forests throughout the Andes Mountains, where they occur from 0 to ~650 m.a.s.l. [20, 21]. In addition, beavers also prefer areas that are not too sloped (>15°), and avoid semi-arid conditions (>600 mm.yr^-1^ EVTP). Pietrek and González-Roglich [36] also noted ecological thresholds in the steppe ecosystem, where beavers probably changed their habitat use patterns as a trade-off for the increased density. In the Northern Hemisphere, it was also noted that the habitat requirements largely determined the distribution and abundance of this species [37], where beavers were associated with forests and some open ecosystems [38]. These findings agree with studies that suggest that beaver habitat use is determined by more than just trees or water [37, 39, 40]. In the archipelago, although the NPP result was linear (more beaver dams in more productive ecosystems), beaver dams were also found in ecosystems with very low productivity (300 gC m^2^ yr^-1^). It is known that NPP is mainly related to temperature and precipitation, which determines the natural macroscale environments in southern Patagonia [41]. In addition, higher temperatures can lead to longer growing seasons and more significant amounts of sunlight in vegetation zones, which could lead to an increase in beaver dams as environments shift from pastures to shrubs and from bushes to forests and peatlands [42], which could be optimal for beavers.

### Beaver dam density variation across environmental gradients

The beaver dam densities have been shown to vary with the geographic, climatic and topographic gradients across the archipelago. Beaver dams were differentially distributed indifferent environmental conditions, so they might not have established a unique equilibrium with the surrounding habitat. Notably, low dam density values occupied the entire dispersion area of the factors (dam density patterns were shown by the multivariate analysis); that is, a low occurrence of beavers might occur in any situation. The distribution of dam density was not necessarily associated with the vegetation zone, but there was a higher density in those places where there was more forest. In fact, the areas that were not invaded were in the extreme vegetation zones (e.g., there are not high densities in tundra or steppe). Certainly, the climatic and topographic characteristics defined the vegetation zones [19–21]; therefore, the precipitation and EVTP gradients were also correlated with dam densities, as well as with the gradient between the productivity and elevation (more elevation, less NPP). The beaver dam densities in the different vegetation zones, mainly those in the mixed-evergreen and deciduous forests, can be further explained by the natural conditions that this vegetation promotes (e.g., wood resources) [5], while the open ecosystem types, such as steep and tundra, or those sparsely covered by forest, such as the ecotone, showed the well-known demand of beavers for timber [3]. It is possible, therefore, that in the ecotone, the plasticity of some native tree species may play a fundamental role in beaver presence. For example, the deciduous *N*. *antarctica* species had high genomic plasticity, which allowed it to occupy extreme sites near to steppe pastures or much more humid areas, such as peatland [43]. Unfortunately, for this study, detailed information about the species composition (e.g., understory plants) or ecosystem types (e.g., shrubland or grassland types) was not available for the whole archipelago at a local level, which limited larger scale knowledge about beaver preferences. The shrub vegetation of the steppe and ecotone may provide enough material for dam construction to all beaver presence in steppe [36] or even in habitats such as tundra [29], which would suggest that vegetation areas without forest can provide substitute environments. In the steppe, Pietrek and González-Roglich [36] showed that beavers first settled in optimal environmental conditions (e.g., watercourses in canyons) and then use other habitats that were initially less preferred, as the numbers of beaver ponds in the study area increased. The temperature affected the beaver dam density change, and its suitability at temperatures below <5°C was less than that at >5°C. Precipitation was not that pronounced, and there was a trend of increase and decrease at 475 mm year^-1^ to 880 mm year^-1^, respectively. These different responses could be more associated with inner landcover variations and mountain areas than with the effects of a zone of influence in maritime areas [44]. Therefore, the climatic conditions likely deflect the beavers to the south in the tundra and the north in the steppes, causing the regional distribution of beavers in the binational forested invasion zone between 54°-55° S to 66°-68° W (Fig 4). However, since the riparian areas of forests play an essential role in beaver biology [2, 7], invasion could be more associated with higher NPP conditions, which shows that the only path for survival is to spread to another area (e.g., the continental mainland). Topography was also a stressor in our study. The elevation showed a positive trend up to the tree line, from 180 to ~400 m.a.s.l. The adverse conditions at low elevations from 0–180 m.a.s.l. were explained by the geophysical characteristics of the steppe and tundra. There were many beaver dams at slopes between 4.5° and 11°, which was explained by the suitable conditions for the building of dams [2]. Henn et al. [12] noted that these characteristics could be most prevalent in mountainous and forested areas.

Biological invasions by non-native species are predicted to increase because of climate change [45]. The reported environmental conditions and the observed beaver preferences suggested that climate change [46] may favor the drivers of beaver invasion (e.g., more NPP could favor an increase in beaver dam density in some ecosystems). For the whole archipelago, an increase in temperature and precipitation was predicted [41]. Earlier reports in the archipelago indicated that the beavers moved to pristine areas and propagated until every natural resource was consumed [9, 14]. Our study showed that beavers overcame sea distances (e.g., Dawson Island, at ±20 km from the main island), and they may have already crossed into pristine areas (e.g., Cape Horn Biosphere Reserve, at ±30 km from the nearest invaded area in Navarino Island). All of this information can be a unique tool for mapping and prioritizing sites for conservation [17] and may be used to design programs that highlight areas where the species can increase their invasion. Our work may provide relevant and comprehensive information for the biogeographical management of invasive species in the archipelago by international agencies such as the Global Environment Facility (GEF), as well as reintroduced species programs in the Northern Hemisphere.

## Conclusions

Here, we presented a density map of beaver dams for the entire Tierra del Fuego archipelago that explained the changes across the landscape in relation to different environmental factors. We made it approachable with the use of simple geoprocessing tools, combined geographic, climatic and topographic factors to create a weighted overlay with the dam density and identified the optimal thresholds. As a result, detailed environmental and non-border notions of their distribution and density throughout the archipelago were presented. The studied factors played a crucial role in the beaver spread. Although the archipelago contains optimal conditions that may facilitate the invasion of beavers, our findings suggested that the invasion of beavers is a phenomenon that has marked preferences and is influenced by the most productive vegetation zones. The single most crucial factor for this result was that beavers were looking for particular environmental conditions in the landscape. These results suggested that above certain thresholds, the beaver dam density increased significantly, e.g., positive ecological responses suggested that the beavers were not only driven by forests but also primarily by the availability of high-productivity ecosystems across the entire geographical range of the archipelago. Considering beaver plasticity, its environmental preferences and distribution status, the threat from beavers may increase with climate change (e.g., in ecosystems with higher NPP), especially in non-invaded areas (e.g., the continental mainland). In future studies (e.g., management and/or control strategies), distribution data (beaver density map) and defined habitat requirements for invasive beavers (e.g., the optimal conditions for beavers) can be used to develop habitat suitability models to anticipate potential invasions and attempt to stop their spread.

## Supporting information

S1 Table Confusion matrix for the calculated error in beaver dam counting based upon the differences between the marked visual survey and the field verified dam location. (DOCX)
