# Supplementary material for: Mapping the status of the North American beaver invasion in the Tierra del Fuego archipelago
Source: PLoS One. 2020 Apr 24;15(4):e0232057. doi: 10.1371/journal.pone.0232057 (PMC7182182; doi:10.1371/journal.pone.0232057)
Supplement: S1 Table — (DOCX) [file pone.0232057.s001.docx]

**S1 Table. Confusion matrix for the calculated error in beaver dam counting based upon the differences between the marked visual survey and the field verified dam location.**

|  |  | **Field data** |  |  |
| --- | --- | --- | --- | --- |
|  |  | **Dam** | **No-Dam** |  |
| Basemaps | Dam | 317 | 42 | 359 |
|  | No-Dam | 42 | 318 | 360 |
|  |  | 359 | 360 | 719 |
